# COVID-19 in Nursing Homes: The Problematic Management of Residents Without Positive COVID-19 RT-PCR

**DOI:** 10.34172/ijhpm.2020.101

**Published:** 2020-06-10

**Authors:** Frédéric Bloch

**Affiliations:** ^1^Department of Geriatric Medicine, University Hospital of Amiens-Picardie, Amiens, France.; ^2^Laboratory of Functional Neurosciences (EA 4559), University of Picardie Jules Verne, Amiens, France.

## Dear Editor,

Since December 2019, a pandemic linked to the new coronavirus severe acute respiratory syndrome coronavirus 2 (SARS-CoV-2), now known as coronavirus disease 2019 (COVID-19), has spread throughout the world. Subjects with COVID-19 may be asymptomatic or present with fever, cough, dyspnea and around 15% may develop acute respiratory distress syndrome. Because older adults are among the most at risk of developing these complications,^1^ several recommendations have been established to better protect this group against the risk of contagion. It is all the more true for older adults living in nursing homes (NH), since they form the most vulnerable group, with comorbidities and dependency. Very early on in the lock-down, therefore, all residents were confined and visiting was severely restricted, on the recommendation of national geriatric societies, in addition to other barrier measures.^2,3^

It was also suggested that, in the event of the appearance of suspicious symptoms in one of the residents, a reverse transcriptase polymerase chain reaction (RT-PCR) must be undertaken to confirm or otherwise the diagnosis of COVID-19. In the case of a positive COVID-19 RT-PCR, the creation of dedicated wings within the NH were recommended for these residents with confirmed or probable COVID-19.^4^ If these recommendations for the management of residents with positive COVID-19 RT-PCR seem obvious to contain the spread of the virus in such structures, it is not the same for those without positive COVID-19 RT-PCR.

Creating a ‘confirmed COVID-19’ sector therefore requires relocating other residents, interchanging them in order to have confirmed or probable COVID-19 residents on one wing and others residents in another in an attempt to keep non-COVID-19 residents in separate areas. But it is not that simple. Indeed, a resident without positive COVID-19 RT-PCR is not a non-COVID-19 resident.

Atypical symptoms of COVID-19 in older adults have been described from feedback of NH with early virus infections. This atypical presentation includes diarrhoea, delirium, falls, fever and lymphopenia. These symptoms are distant from the more classic respiratory signs initially described and may be interpreted as a resident being negative for COVID-19.

Performing RT-PCR systematically for atypical presentations or asymptomatic residents does not furthermore give an answer with certainty and has to be considered as not useful. Indeed, it has been reported that a significant rate of false negative of around 30% is frequent in the result of RT-PCR for COVID-19 diagnosis.^5^ This source of misunderstanding can lead to a risk of improper reassurance and to an inappropriate reduction of protective and barrier procedures, which can be dramatic in the case of wandering residents. This risk is also found for the two-thirds of subjects whose RT-PCR is truly negative. As a matter of fact, a negative test in an asymptomatic resident does not mean that he will not be positive as long as he is in the incubation phase which lasts an average of 14 days.

It is therefore preferable to consider all residents, whether symptomatic or not and whatever their symptomatology, as a possible COVID-19 case rather than trying, with a relatively low success rate, to determine among residents who is COVID-19 positive and who is COVID-19 negative. It is still possible to remove and isolate the confirmed or probable COVID-19 residents from their unit if the physical layout of the units enables it. But, to avoid interchanges from one unit to the other of residents of whom we do not know the COVID-19 status, this must been done without moving the asymptomatic residents without positive COVID-19 RT-PCR. These measures prevent bringing together residents without positive COVID-19 RT-PCR with the risk to put truly negative COVID-19 residents in contact with false negative or future positive residents as an asymptomatic resident even without positive COVID-19 RT-PCR cannot be considered as a negative COVID-19.

Measures in strict compliance with protective measures must be defined for each unit and each room maintaining the containment for all residents either in single room or in their unit while maintaining barrier gestures. The sectoring of nurses, assistant nurses and professionals directly in contact with the residents in dedicated units rather than dispatching them in all the units may also represent an effective measure to reduce the spreading of the virus.

It is the multiplication of these barrier measures that will be the best weapon in the fight against the virus. This is the price to be paid to prevent the entry of COVID-19 into NH and limit its spread.

## Ethical issues

Not applicable.

## Competing interests

Author declares that he has no competing interests.

## Author’s contribution

FB is the single author of the paper.
